# Level of clinical evidence presented at the open and closed American Shoulder and Elbow Surgeons annual meeting over 10 years (2005–2014)

**DOI:** 10.1186/s12891-016-1334-y

**Published:** 2016-11-14

**Authors:** Jeffrey Kay, Muzammil Memon, Darren de SA, Nicole Simunovic, George S. Athwal, Asheesh Bedi, Olufemi R. Ayeni

**Affiliations:** 1 Michael G. DeGroote School of Medicine, McMaster University, Hamilton, ON Canada; 2 Division of Orthopaedic Surgery, Department of Surgery, McMaster University, 1200 Main Street West, 4E15, Hamilton, L8N 3Z5 ON Canada; 3 Department of Clinical Epidemiology and Biostatistics, McMaster University, Hamilton, ON Canada; 4 Roth|McFarlane Hand and Upper Limb Center, St Joseph’s Health Care, Western University, London, ON Canada; 5 Sports Medicine and Shoulder Service, Department of Orthopaedic Surgery, University of Michigan, Ann Arbor, MI USA

**Keywords:** LOE, Scientific meeting, Shoulder and elbow, Quality

## Abstract

**Background:**

The American Shoulder and Elbow Surgeons (ASES) annual scientific meetings are premier forums whereby orthopaedic surgeons are informed of the latest research advances in shoulder and elbow surgery. The purpose of the present study was to assess the Level of evidence (LOE) in the clinical papers presented at both the open and closed ASES annual scientific meetings from 2005 to 2014. Secondarily, the study evaluated whether there were any changes in the distribution of LOE over this period of time.

**Methods:**

Two reviewers independently evaluated the abstracts of 532 paper presentations at either the open or closed ASES annual meetings. The independent reviewers first screened the abstracts for clinical evidence and excluded cadaveric, biomechanical, technique, and review studies. The included abstracts were then independently graded for methodological quality using LOE from Level I (highest quality) to IV (lowest quality) based on the classification system created by The American Academy of Orthopaedic Surgeons (AAOS).

**Results:**

Overall, 421 presentations were included and graded for LOE. In general, 17% of the presentations were graded level I; 15% level II; 25% level III; and 43% assigned a LOE of IV. Chi-square analysis demonstrated a statistically significant improvement in the LOE of presentations at the open and closed ASES meetings combined (*p* = 0.028) between the years 2005 and 2014. In particular, the proportion of presentations graded as level IV significantly decreased over this period (*p* = <0.001).

**Conclusions:**

While most presentations at the ASES annual scientific meetings were of lower LOEs the percentage of level I evidence is greater than that reported at other Orthopaedic meetings. There has been a significant improvement in the LOE of clinical research at open and closed ASES meetings from 2005 to 2014. Specifically, the proportion of level IV studies have dramatically decreased over time.

## Background

The American Shoulder and Elbow Surgeons (ASES) is a leading subspecialty association comprised of shoulder and elbow surgeons and focuses on promoting the highest quality of care available. The ASES holds two meetings each year (closed for members, and open for both members and non-members). These meetings act as important sources of information for all Orthopaedic surgeons and are particularly influential on those who are focusing on the shoulder or elbow joints. Research findings from presentations at these meetings may have a direct impact on clinical decision-making or even on health policies. Thus, it is critical that the research presented at these meetings is of quality that is near the top of the hierarchy of evidence. Sackett and colleagues introduced the important ideas of Evidence-Based Medicine which emphasizes the importance of research from study designs of high methodological quality in clinical decision making [1, 2]. One method of classifying the methodological quality of a study is by using Level of evidence (LOE). Such LOEs (level I to level IV) are assigned to a study based on the methodological design of the study with high quality randomized controlled trials (RCT) grades as level I and case report or case series graded as level IV. It is thought that studies with higher quality methodology can be applied more reliably to clinical practice. A standard approach for grading Orthopaedic research has been created by The American Academy of Orthopaedic Surgeons (AAOS) which is an updated version of the system originally described by *The Journal of Bone and Joint Surgery* [3].

The quality and type of presentations at scientific meetings may be effected by different factors. For example, the inherent nature of Orthopaedics may inhibit the production of high quality evidence with rigorous methodology, particularly when studying new procedures. The incidence and applicability of elbow and shoulder arthroscopy have increased since the turn of the millennium and their use has recently expanded and are now indicated for some fractures as well [4]. Furthermore, under the influence of EBM, many Orthopaedic journals began introducing LOEs to all publications between 2003 and 2005. Another important consideration pertains to the difference between the open and closed meetings. The open meetings typically contain presentations of more common and studied topics, and therefore often have immediate clinical implication. On the other hand, newer topics that are in the critical review process are often presented at the closed meetings. The purpose of the present study was to evaluate the LOE of the papers presented at both the open and closed ASES annual scientific meeting between the year 2005 and 2014. Secondarily, this study assessed whether there were any changes in the distribution of LOE over this period of time.

## Methods

### Study eligibility

The methodology used to assess the quality of presentations at ASES meetings in this study are similar to those used to assess the quality of presentations at the International Society for Hip Arthroscopy (ISHA) meeting in a previous study [5]. The inclusion criteria were clinical papers presented at the ASES annual open and closed meeting between 2005 and 2014. Any trial or observational study with direct communication between human subjects and an investigator and was considered to be a clinical study. The exclusion criteria were cadaveric studies, biomechanical studies, technique presentations, and expert panel discussions.

### Screening and grading

The abstracts for papers presented at several of the open and closed ASES annual meetings have been electronically published and made available through *The Journal of Shoulder and Elbow Surgery* (JSES). The ASES was contacted to inquire about abstracts that were not published. The abstracts of available presentations were screened independently by two reviewers. The included abstracts were then graded for LOE (Level I to IV) by two independent reviewers using the AAOS classification scheme [3]. If there were any disagreements, these were discussed by the two reviewers at the end of the initial assessment until they reached a consensus. A senior author was consulted as necessary for disagreements.

### Data extraction and statistical analysis

Data that was abstracted from the included presentations include: study type, sample size, LOE, and study location. Microsoft Excel 2013 (Microsoft, Redmond, WA) was used to record extracted data. Kappa (κ) was calculated for both the screening stage and the presentation evaluation stage to evaluate the inter- reviewer agreement. A priori categorization of the level of agreement was as follows: κ of 0.20 or less was considered slight agreement; κ of 0.21 to 0.60, moderate agreement; and κ of 0.61 or greater, substantial agreement. For all meetings included in the analysis, the frequency of each LOE was calculated by dividing the number by the total number of included presentations for that meeting. Non-random statistical changes in the LOE distribution over time were evaluated using Pearson Chi-square analyses. While these tests do not tell us about trend, they do provide important information regarding the change in LOE over time. A significance level of *p* ≤ 0.05 was used for the general analyses. However, when all four LOEs were evaluated separately, this threshold was adjusted to 0.0125 using the conservative Bonferroni correction for multiple tests. Minitab ® statistical software version 17 (Minitab Inc., State College, USA) was used for all statistical calculations.

## Results

Four hundred twenty-one of the 532 presentations available online between 2005 and 2014 met the inclusion criteria and were assessed for LOE. There was no data available on presentations from the open meeting in 2007 and on the closed meeting in 2010, 2011 and 2013, as the abstracts from these meetings were not published, and they could not be obtained through contacting the ASES administrators. The agreement between reviewers was considered to be substantial for both abstract screening as well as LOE evaluation with κ (95% confidence intervals [CI]) values of 0.98 (0.93, 1.00) and 0.85 (0.82, 0.88), respectively. In general, 17% (95% CI, 13 to 21%) of the presentations were graded as level I evidence, 15% (95% CI, 12 to 18%) level II evidence, 25% (95% CI, 21 to 29%) level III evidence and 43% (95% CI, 38 to 48%) level IV evidence with a mean LOE of III (2.93) over the 10 years that were analyzed.

The 421 presentations that were evaluated had a mean sample size of 363 (standard error of the mean [SEM] 172) subjects. Therapeutic studies were the most commonly presented study design overall (64%, 95% CI: 59 to 69%), however, there was variation in the proportion of therapeutic studies for each LOE. For example, only 41% of all level II studies in comparison to 92% of level IV studies were therapeutic. Prognostic studies were the next most frequently presented study (28%, 95% CI: 24 to 32%) while diagnostic and economic studies comprised only 5% (95% CI, 3 to 7%) and 1% (95% CI 0 to 1%) of all presentations, respectively. There were a total of 15 different countries recorded as the country of record for the primary authors, although only 83 of 421 were from outside of the USA.

**Fig. 1:**
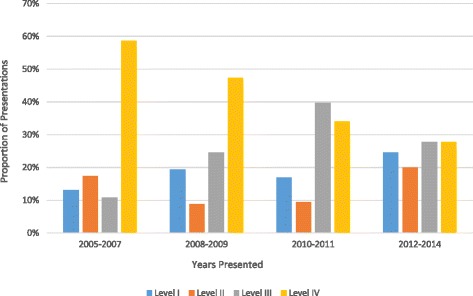
The percentage of scientific clinical presentations by LOE and years of presentation for the open ASES meetings

**Fig. 2:**
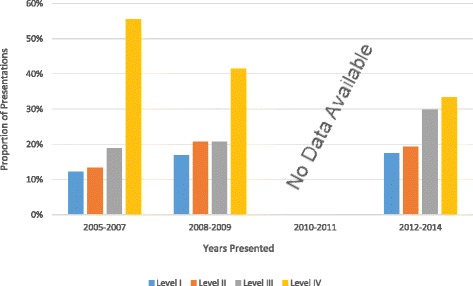
The percentage of scientific clinical presentations by LOE and year of presentation for the closed ASES meetings

Comparing all abstracts presented in the first five years of our analysis (2005–2009) with abstracts over the last five years of the analysis (2010–2014) the percentage of level I (15 to 20%), level II (15 to 17%) and level III (19 to 32%) evidence increased while the percentage of level IV evidence (51 to 31%) decreased. The percentage of papers presented over time is displayed in Figs. 1 and 2 for the open and closed meetings respectively.

When comparing the meeting types, the open meeting had a higher percentage of level I (19% vs. 15%) and a lower percentage of level IV presentations (41% vs. 46%) than the closed meeting. The closed meeting had a higher percentage of level II (17% vs. 14%) and a lower percentage of level III presentations (26% vs. 23%). There was no significant difference between the LOEs presented at the open meeting in comparison to the closed meeting (*p* = 0.805).

Due to the small annual sample size at each meeting (20–40 presentations), the chi-squared analysis resulted in few significant changes when comparing meetings individually. The overall association between LOE and year presented for presentations at the open (*p* = 0.089) and closed (*p* = 0.127) meetings alone were not significant. The open and closed meetings individually showed no significant changes in level I (*p* = 0.405, *p* = 0.499), level II (*p* = 0.465, *p* = 0.342) or level III (*p* = 0.143, *p* = 0.170) evidence over the years 2005–2014. While there were no significant changes in level IV evidence at the closed meeting (*p* = 0.028) there was a significant (*p* = 0.0120) decrease in level IV evidence presented at the open meetings between 2005 and 2014.

There was a significant, non-random improvement in LOE overall for presentations presented at the open and closed meeting combined (*p* = 0.028) over the years 2005–2014. Focused analysis of each LOE revealed no significant changes in level I evidence (*p* = 0.770), level II evidence (*p* = 0.421) or level III evidence (*p* = 0.046), but there was a significant decrease in level IV evidence (p < 0.001).

## Discussion

The ASES annual scientific meetings are important venues that focus on the presentation of high quality and evidence-based outcome measures for the most important procedures addressing injuries or disease of the shoulder and elbow.

Overall, the presentations over the past ten years at ASES meetings predominantly used lower quality study designs, with 68% of all presentations graded as either level III or IV evidence. The most common presentations were level IV studies, which encompassed 41% of paper presentations from the open meeting and 46% of paper presentations from the closed meeting between 2005 and 2014. Level IV studies are mostly case series, which describe outcomes from a single cohort of patients without comparison to a control group. The lack of a control group affords level IV studies with inherent sources of bias including: measurement bias, selection bias, and bias from confounding factors that are unaccounted for. As such, the attendees of these meetings should avoid using the outcomes generated from such studies as the sole basis of treatment decisions. While research of lower methodological quality may not always have direct clinical relevance, these studies are important sources of information that can be used to generate hypotheses to direct the focus of future studies with improved study designs [6].

Between 2005 and 2014, there was a significant decrease in the presentation of level IV evidence in both the open and closed annual meetings combined and in the open meeting alone. These changes could indicate an increased understanding and use of new procedures concerning the shoulder and elbow. Due to the aging population, degenerative shoulder problems such as osteoarthritis and rotator cuff disease are becoming increasingly common [7]. The use of shoulder arthroplasty has increased substantially since the United States Food and Drug Administration approved the use of reverse total arthroplasty in 2003 [8]. This procedure has been a very important contribution for treatment of conditions such as rotator cuff insufficiency, arthropathy and some fractures [7]. The use of elbow arthroscopy was initially described as unfeasible in 1931, however newer technology and a better understanding of the anatomy of the elbow has since allowed an increase in the prevalence of arthroscopic elbow surgery [4]. As data is compiled from early research evaluating these new procedures, the production of research with improved study designs becomes progressively more feasible. Furthermore, many Orthopaedic journals including the Journal of Bone and Joint Surgery, American Journal of Sports Medicine, and JSES have required and included the LOE as part of the publication of research manuscripts. Not only do published LOE allow clinicians to understand the context with which to interpret a study, but it also provides Orthopaedic surgeons a greater awareness of the importance of studies with higher methodological quality or LOE [9]. Most Orthopaedic journals, including the JSES, have demonstrated increased production of publications with level I and II evidence since the introduction of LOE in these journals [10]. Therefore, we could attribute the improvement in the methodological quality of research at ASES meetings to an increased quantity of research submitted in addition to the influence of EBM, facilitated partially by the inclusion of LOE in most Orthopedic journals at the beginning of our study period.

Although the statistical analysis did not indicate a significant change in the proportion of level I studies between 2005 and 2014, the relatively small sample size of level I evidence may have contributed to the negative finding. The percentage of level I evidence increased from 15% before 2010 to 20% after 2010. Of the 17% of studies that were classified as level I, 57% were therapeutic indicating that 10% of all studies were RCTs. The percentage of level I evidence at ASES meetings between 2005 and 2014 represents a much higher number than that presented at the AAOS (7% in 2010) meeting and even greater than the percentage of level I and II presentations combined at the Pediatric Orthopaedic Society of North America (POSNA) meetings (14% over 2001, 2002, 2007 and 2008) [11, 12]. The proportion of level I research is especially impressive considering the recognized difficulty in performing a randomized study with surgical interventions. Some key challenges inherent to surgical trials include patients’ reluctance to randomization as well as the difficulty blinding surgeons to the interventions [13]. These challenges limit the number of RCTs conducted, but also impact the quality of those produced as Bhandari et al. reported that the majority of randomized trials published in *The Journal of Bone and Joint Surgery* did not report the use of key methodological features such as proper allocation concealment, exclusion of patients, and blinded assessment of outcomes [14]. RCTs studying elbow pathology in particular, are not of the highest quality and have not shown an improvement in their quality in recent years [15]. On the other hand, McCormick et al. have reported that, according to the Jadad score, RCTs studying rotator cuff pathology are of high quality [14, 16]. Although there are difficulties conducting RCTs in surgery, Farrokhyar et al. have reported that RCTs for novel surgical interventions are important and can be produced if the feasibility is properly assessed initially and close attention is addressed to specific methodological details [12].

Overall, statistical analyses indicate a significant improvement in the LOE of presentations at the open and closed annual ASES meetings from 2005 to 2014. The average LOE during the period between 2005 and 2009 was 3.07 and improved to 2.75 in the period between 2010 and 2014. Voleti and colleagues evaluated the poster and paper presentations at the AAOS meetings and demonstrated significant improvements in the average LOE between 2001 (3.46) and 2010 (2.88) [11].

The program committee for ASES meetings diligently screen submitted abstracts and include only presentations of the highest quality, however, the quality of abstracts that are being submitted is often the limiting factor to such selection. Therefore, the changes identified in the present study are likely indicative of an improvement in the methodological quality of the research being submitted for presentation at these meetings. One method that can be used to educate the audience as well as the submitting authors on the importance of high methodological quality is having authors include the LOE of their study accompanying the abstract. However, Shmidt et al. studied presentations for which authors provided a LOE at AAOS meetings and have reported that authors tend to rate their own studies with a higher LOE than an independent reviewer [17]. Therefore, if LOEs are to be included with meeting presentations, it may be beneficial to have these verified by an independent reviewer.

A strength of this study is that it is the first, to the best knowledge of the authors, to evaluate the methodological quality of the research presented at the ASES annual scientific meetings. There was high agreement amongst the reviewers in terms of the evaluation of LOEs, indicating that the evidence was categorized consistently. Limitations of this study include the availability of abstracts (no abstracts from the years 2007, 2010, 2011 and 2013) and the limited methodological information available based on the 300 word abstract alone. Missing data may have affected the statistical tests assessing the methodological quality over time.

## Conclusions

The ASES annual meeting is at the forefront in presenting the latest research in the arenas of shoulder and elbow surgery and therefore the presentations at these meetings may act as a surrogate marker for the latest research in these fields. While most presentations at the ASES annual scientific meetings were of lower LOEs the percentage of level I evidence is greater than that reported at other Orthopaedic meetings. There has been a significant improvement in the LOE of clinical research at open and closed ASES meetings from 2005 to 2014. Specifically, the proportion of level IV studies have dramatically decreased over time.

## Abbreviations

AAOS: American Academy of Orthopaedic Surgeons
ASES: American Shoulder and Elbow Surgeons
EBM: Evidence-Based Medicine
JSES: *The Journal of Shoulder and Elbow Surgery*
